# Chloroplast genome for *Crataegus pinnatifida* (Rosaceae) and phylogenetic analyses with its coordinal species

**DOI:** 10.1080/23802359.2019.1667273

**Published:** 2020-05-18

**Authors:** Shui-Lian He, Jing Xie, Yang Yang, Yang Tian

**Affiliations:** a College of Horticulture and Landscape, Yunnan Agricultural University, Kunming, Yunnan, China; b Yunnan Key Laboratory of Biomass Big Data, Yunnan Agricultural University, Kunming, Yunnan, China; c College of Science, Yunnan Agricultural University, Kunming, Yunnan, China

**Keywords:** *C. pinnatifida*, medicinal and edible plant, chloroplast genome, phylogeny

## Abstract

*Crataegus pinnatifida* is an important medicinal and edible plant. Now, the complete chloroplast (cp) genome of *C. pinnatifida* was assembled and annotated. The cp genome of *C. pinnatifida* was 159,898 bp and contained two short inverted repeat regions (26,540 bp) which were separated by a small single copy region (19,219 bp) and a large single copyregion (87,599 bp). The cp genome encodes 109 unique genes, including 75 protein-coding genes, 30 transfer RNA genes and 4 ribosomal RNA genes. The topology of the phylogenetic tree showed that *C. pinnatifida has a close relationship with species Eriobotrya, Sorbus, Pyrus, Mulus, and Chaenomeles*.

*Crataegus pinnatifida* Bunge, (Hawthorn) belongs to the family Rosaceae and is a widespread fruit tree in China. The fruits are used for food and medicinal purposes. The leaves, fruits, roots, and twigs of hawthorn contain many nutrients, including proteins, fats, dietary fibre, vitamins, flavones, and many minerals (Wang et al. 2011; Rodrigues et al. 2012; Yang and Liu 2012). In order to clarify the taxonomical position of *C. pinnatifida* in Rosaceae, We applied the Illumina technology to sequence, assemble and annotate the whole chloroplast genome of *C. pinnatifida*. The resultant data have been made publicly available as a resource for genetic information for *Mentha* species, and will provide a valuable plastid genomic resource for the future genetic and phylogenetic studies about *C. pinnatifida.*

The fresh leaves of *C. pinnatifida* were collected from the field of Kunming (25.20°N, 102.86°E). The voucher specimen was deposited at Herbarium of Yunnan Agricultural University (No. 2019HSL002). Total genomic DNA was isolated from fresh leaves using a DNeasy Plant Mini Kit (QIAGEN, Valencia, California, USA) according to the manufacturer’s instructions to construction chloroplast DNA libraries. The Illumina sequencing was conducted by Biomarker Technologies Inc. (Beijing, China). Resultant clean reads were assembled using GetOrganelle pipeline (https://github.com/Kinggerm/GetOrganelle). The genome was automatically annotated by using the CpGAVAS pipeline (Liu et al. 2012) and start/stop codons and intron/exon boundaries were adjusted in Geneious R11.0.2 (Biomatters Ltd., Auckland, New Zealand). All the contigs were checked against the reference genome of *Malus trilobata* (NC035671).

The complete chloroplast genome of *C. pinnatifida* was 159,898 bp in length (Genbank accession number: MN102356). It was the typical quadripartite structure and contained contained two short inverted repeat (IRa and IRb) regions (26,540 bp) which were separated by a small single copy (SSC) region (19,219 bp) and a large single copy (LSC) region (87,599 bp). The cp genome encodes 109 unique genes, including 75 protein-coding genes, 30 transfer RNA (tRNA) genes and 4 ribosomal RNA (rRNA) genes. Twenty-one gene species are partially or completely duplicated, including ten PCG (*ndhB*; *ndhF*; *rpl2*; *rpsl23*; *rps12*; rps19; *rps7*; *ycf1*; *ycf2*), seven tRNA (*trnI-GAU*, *trnA-UGC*, *trnL-CAA*, *trnI-CAU*, *trnR-ACG*, *trnV-GAC*, *trnN-GUU*) and all four rRNA (4.5S, 5S, 16S & 23S rRNA). The overall GC content of the cp genome was 36.6%, while that of LSC, SSC and IR regions was 34.4%, 30.4% and 42.6%, respectively.

A total of 30 cp genome sequences were selected to infer the phylogenetic relationships among the main representative species of Rosaceae with *Magnolia yunnanensis* (*NC024545,* Magnoliaceae) as outgroup. The combined datasets based on plastid genomes were aligned by MAFFT v7.307 (Katoh and Standley 2013). A neighbour-joining (NJ) phylogenetic tree was constructed in Geneious 11.1.5 (Kearse et al. 2012) with the Tamura-Nei genetic distance model, and a total of 1000 bootstrap replicates were performed. The topology of the phylogenetic tree showed that *C. pinnatifida has a close relationship with species Eriobotrya, Sorbus, Pyrus, Mulus, and Chaenomeles* (Figure 1). The complete cp genome information reported in this study will be a valuable resource for future studies on genetic diversity, taxonomy and phylogeny of the Rosaceae.

**Figure 1.**
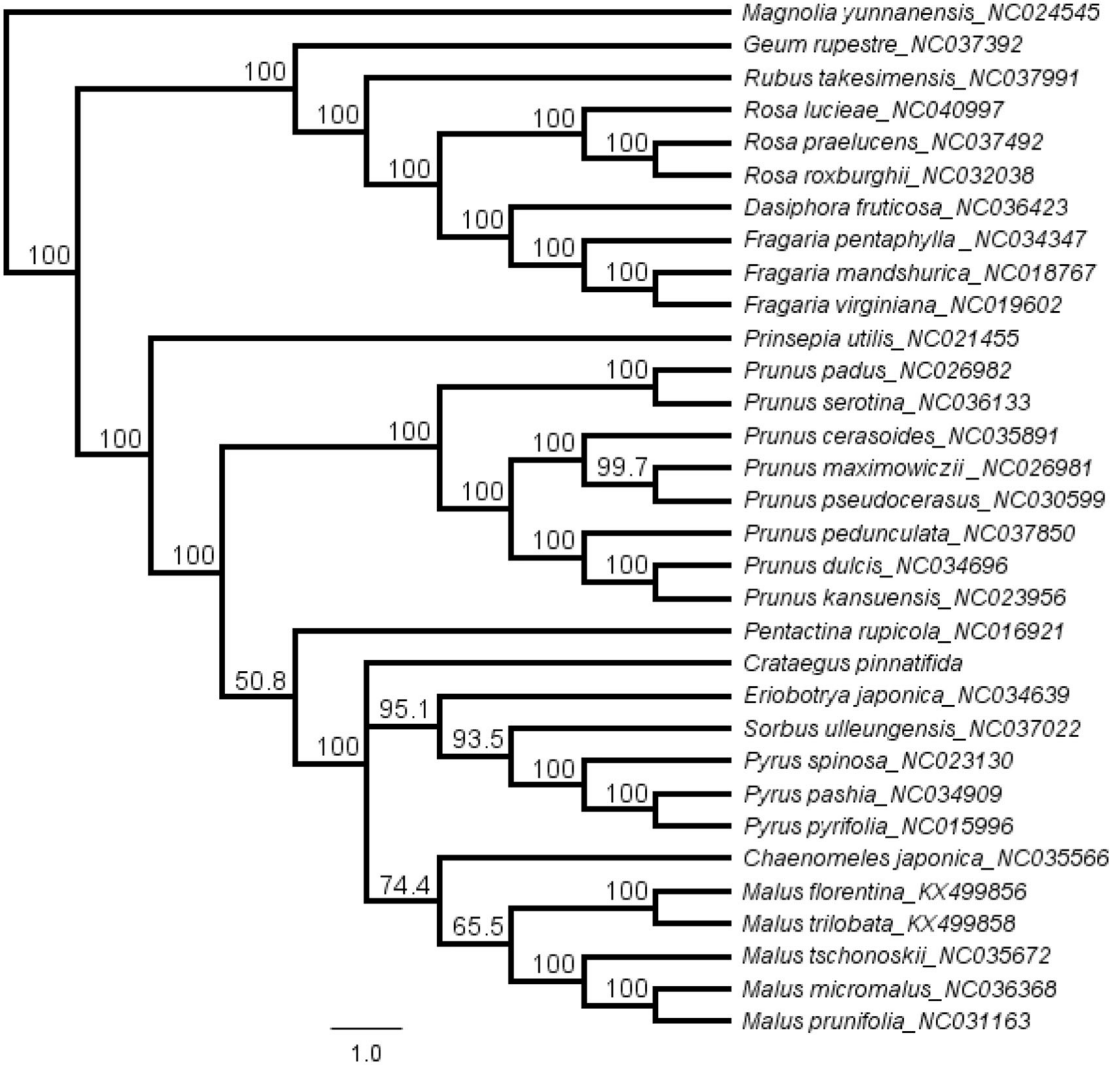
The neighbor-joining (NJ) phylogenetic tree based on 32 complete chloroplast genome sequence. Numbers at the right of nodes are bootstrap support values.

## References

[CIT0001] Katoh K, Standley DM. 2013. MAFFT multiple sequence alignment software version 7: improvements in performance and usability. Mol Biol Evol. 30(4):772. 23329690 10.1093/molbev/mst010 PMC3603318

[CIT0002] Kearse M, Moir R, Wilson A, Stones-Havas S, Cheung M, Sturrock S, Buxton S, Cooper A, Markowitz S, Duran C, et al. 2012. Geneious Basic: an integrated and extendable desktop software platform for the organization and analysis of sequence data. Bioinformatics. 28(12):1647. 22543367 10.1093/bioinformatics/bts199 PMC3371832

[CIT0003] Liu C, Shi L, Zhu Y, Chen H, Zhang J, Lin X, Guan X. 2012. CpGAVAS, an integrated web server for the annotation, visualization, analysis, and GenBank submission of completely sequenced chloroplast genome sequences. BMC Genomics. 13(1):715. 23256920 10.1186/1471-2164-13-715 PMC3543216

[CIT0004] Rodrigues S, Calhelha RC, Barreira JCM, Dueñas M, Carvalho AM, Abreu RMV, Santos-Buelga C, Ferreira ICFR. 2012. Crataegus monogyna buds and fruits phenolic extracts: growth inhibitory activity on human tumor cell lines and chemical characterization by HPLC–DAD–ESI/MS. Food Res Int. 49(1):516–523.

[CIT0005] Wang T, An Y, Zhao C, Han L, Boakye-Yiadom M, Wang W, Zhang Y. 2011. Regulation effects of *Crataegus pinnatifida* leaf on glucose and lipids metabolism. J Agric Food Chem. 59(9):4987–4994. 21425878 10.1021/jf1049062

[CIT0006] Yang B, Liu P. 2012. Composition and health effects of phenolic compounds in hawthorn (*Crataegus* spp.) of different origins. J Sci Food Agric. 92(8):1578–1590. 22488722 10.1002/jsfa.5671

